# Triple HER2 Blockade With Trastuzumab, Pertuzumab, and Pyrotinib Versus Dual HER2 Blockade in the Neoadjuvant Treatment of HER2‐Positive Breast Cancer: A Randomized, Phase II Study

**DOI:** 10.1002/mco2.70611

**Published:** 2026-01-18

**Authors:** Jiahui Huang, Haoyu Wang, Yiwei Tong, Jin Hong, Yifei Zhu, Weili Ren, Jing Yu, Haoting Shi, Weiqi Gao, Siji Zhu, Jiayi Wu, Ou Huang, Jing Li, Jianrong He, Weiguo Chen, Yafen Li, Kunwei Shen, Xiaosong Chen

**Affiliations:** ^1^ Department of General Surgery Comprehensive Breast Health Center Ruijin Hospital Shanghai Jiao Tong University School of Medicine Shanghai China; ^2^ Department of Breast and Thyroid Surgery Shangyu People's Hospital Zhejiang China; ^3^ Department of Medical Affairs Jiangsu Hengrui Pharmaceuticals Co., Ltd Shanghai China

**Keywords:** breast cancer, neoadjuvant therapy, HER2 blockade, pathological complete response, pyrotinib

## Abstract

This study aimed to evaluate the efficacy and safety of triple human epidermal growth factor receptor 2 (HER2) blockade with trastuzumab, pertuzumab, and pyrotinib (TPPy) versus dual HER2 blockade with trastuzumab and pertuzumab (TP) in the neoadjuvant treatment of HER2‐positive breast cancer. Patients with stage II–III HER2‐positive breast cancer were randomized (1:1) to receive TPPy or TP alongside weekly nab‐paclitaxel for 12 weeks. The primary endpoint was total pathological complete response (tpCR; ypT0/isN0). Exploratory biomarker and pathway analysis was done to identify patients benefiting from pyrotinib. A total of 109 patients were enrolled, and 108 received treatment: 55 in the TPPy group and 53 in the TP group. The tpCR rate was 65.5% (95% confidence interval [CI]: 51.4%–77.8%) in the TPPy group, and 60.4% (95% CI: 46.0%–73.5%) in the TP group (*p* = 0.585). In the TPPy group, 52 (94.5%) and 23 (41.8%) patients experienced dose interruption and discontinuation, respectively. The most common grade ≥3 adverse events in the TPPy and TP groups were diarrhea (58.1% vs. 0%) and neutropenia (23.6% vs. 15.1%). In conclusion, triple HER2 blockade did not improve tpCR rates compared with dual blockade but was associated with greater toxicity, particularly diarrhea.

## Graphical abstract

Triple HER2 blockade with trastuzumab, pertuzumab, and pyrotinib (TPPy) did not improve total pathological complete response (tpCR) rates or breast pathological complete response (bpCR) rates compared with dual blockade with trastuzumab and pertuzumab (TP), but was associated with greater grade ≥ 3 toxicity, particularly diarrhea.

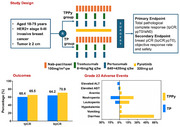

## 1 Introduction

Human epidermal growth factor receptor 2 (HER2)‐positive breast cancer, characterized by overexpression of the HER2 protein or amplification of the HER2 gene, accounts for approximately 15%–20% of all breast cancer cases [1]. In early‐stage HER2‐positive disease, neoadjuvant anti‐HER2 therapy has become a standard treatment approach, with pathological complete response (pCR) recognized as a well‐validated surrogate endpoint for long‐term outcomes such as event‐free survival and overall survival [2, 3].

Dual HER2 blockade with trastuzumab and pertuzumab (TP) combined with chemotherapy is the standard neoadjuvant therapy for early‐stage or locally advanced HER2‐positive breast cancer. Evidence from the NeoSphere and PEONY trials indicates that four cycles of TP plus docetaxel achieve a pCR of approximately 40% in patients with HER2‐positive disease [4, 5]. However, nearly half of these patients fail to achieve pCR following neoadjuvant therapy due to primary or secondary treatment resistance mechanisms. These mechanisms include structural alterations in the HER2 receptor, intrinsic activation of downstream signaling components, activation of alternative pathways by other HER family members, and intercellular changes induced by anti‐HER2 therapies that affect the PI3K pathway [6].

Pyrotinib is an irreversible tyrosine‐kinase inhibitor (TKI) that targets EGFR/HER1, HER2, and HER4 [7]. By covalently binding to the ATP‐binding site of the intracellular kinase domain of HER, pyrotinib can inhibit the autophosphorylation of HER homodimers and heterodimers, thereby blocking the Ras/Raf/MEK/MAPK and PI3K/Akt signaling pathways. Pyrotinib‐containing regimens have demonstrated significant efficacy and favorable safety in multiple pivotal studies and have been approved in China for first‐ and second‐line treatment of HER2‐positive advanced breast cancer as well as for neoadjuvant therapy in early‐stage HER2‐positive breast cancer. In the neoadjuvant PHEDRA study, the addition of pyrotinib to trastuzumab and docetaxel increased the pCR rate from 22.0% to 41.0% [8]. However, despite this notable improvement, a considerable proportion of patients still failed to achieve pCR, highlighting the need for more effective therapeutic strategies. Furthermore, in an in vitro study, the combination of TP with TKIs exhibited synergistic activity in suppressing HER2‐positive breast cancer cell proliferation, which is currently under clinical investigation [9].

Therefore, we conducted the first randomized phase II trial to evaluate the efficacy and safety of triple HER2 blockade with trastuzumab, pertuzumab, and pyrotinib (TPPy) versus dual TP therapy in the neoadjuvant treatment of patients with HER2‐positive breast cancer.

## 2 Results

### 2.1 Enrolled Patients

**FIGURE 1:**
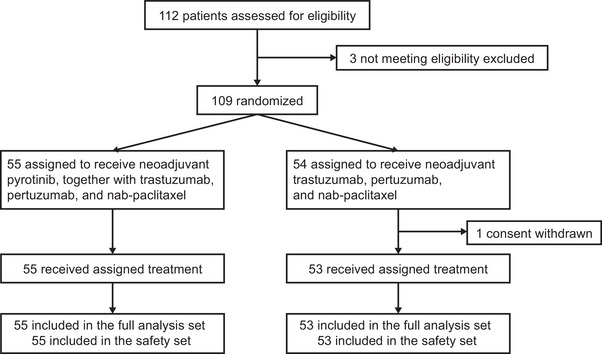
Workflow of the study.

Between November 23, 2020, and April 21, 2023, 109 eligible patients were randomly assigned (Figure 1). One patient withdrew before receiving any study treatment. Among the remaining participants, 53 patients were included in the TP group: 32 (60.4%) were hormone receptor (HR)‐negative and 42 (79.2%) had stage III disease. Fifty‐five patients in the TPPy group received the same regimen with the addition of pyrotinib: 33 (60.0%) were HR‐negative and 46 (83.6%) had stage III disease. Baseline characteristics were well balanced between the two groups (Table 1). After completing neoadjuvant treatment, all 108 patients underwent radical surgery, and histopathological evaluation was performed for each. Adjuvant therapies administered to patients are summarized in Table S1.

**TABLE 1:** Baseline demographics and disease characteristics.

Characteristics, *N* (%)	TPPy group	TP group	*p* value
	(*N* = 55)	(*N* = 53)	
Age			0.720
≤ 50 years	22 (40.0)	23 (43.4)	
> 50 years	33 (60.0)	30 (56.6)	
Menstruation			0.980
Postmenopause	24 (43.6)	23 (43.4)	
Pre/perimenopause	31 (56.4)	30 (56.6)	
BMI, kg/m^2^			0.455
< 24	38 (69.1)	33 (62.3)	
≥ 24	17 (30.9)	20 (37.7)	
Clinical tumor stage			0.581
0–2	34 (61.8)	30 (56.6)	
3–4	21 (38.2)	23 (43.4)	
Clinical node stage			0.214
0	4 (7.3)	5 (9.4)	
1	8 (14.5)	13 (24.5)	
2	22 (40.0)	24 (45.3)	
3	21 (38.2)	11 (20.8)	
Clinical AJCC stage			0.651
II	9 (16.4)	11 (20.8)	
IIIA	25 (45.5)	26 (49.1)	
IIIB–C	21 (38.2)	16 (30.2)	
Estrogen receptor status			0.585
Positive	19 (34.5)	21 (39.6)	
Negative	36 (65.5)	32 (60.4)	
Progesterone receptor status			0.576
Positive	12 (21.8)	14 (26.4)	
Negative	43 (78.2)	39 (73.6)	
Hormone receptor status			0.968
Positive	22 (40.0)	21 (39.6)	
Negative	33 (60.0)	32 (60.4)	
Ki67, %			0.322
≤ 30	27 (49.1)	21 (39.6)	
> 30	28 (50.9)	32 (60.4)	
HER2 IHC			0.920
2+	15 (27.3)	14 (26.4)	
3+	40 (72.7)	39 (73.6)	

Abbreviations: AJCC, American Joint Committee on Cancer; BMI, body mass index; HER2, human epidermal growth factor receptor 2; IHC, immunohistochemistry; TP, trastuzumab, pertuzumab; TPPy, trastuzumab, pertuzumab, and pyrotinib.

### 2.2 Efficacy

**FIGURE 2:**
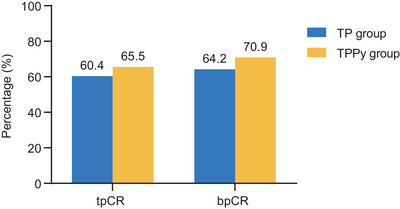
Pathological complete response rate in the TP and TPPy groups. bpCR, breast pathological complete response (ypT0/is); TP, trastuzumab, pertuzumab; tpCR, total pathological complete response (ypT0/isN0); TPPy, trastuzumab, pertuzumab, and pyrotinib.

**FIGURE 3:**
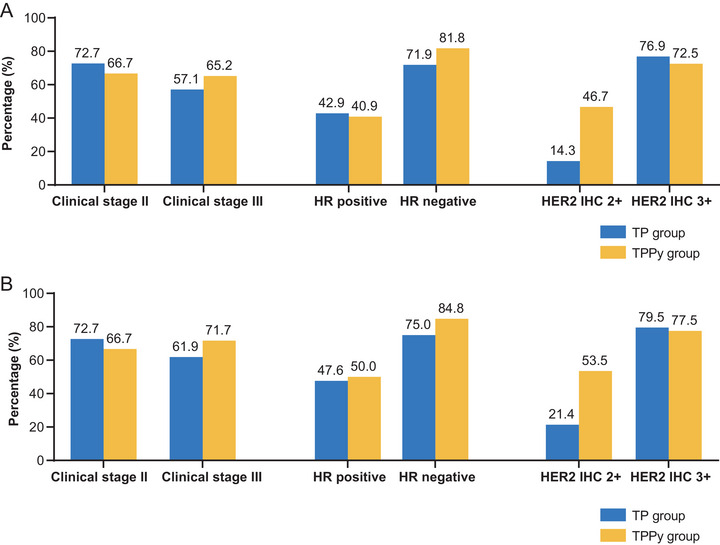
Subgroup analysis of pCR rates in the TP and TPPy groups. (A) The tpCR (ypT0/isN0) rates according to clinical stage, hormone receptor status, and HER2 IHC score. (B) bpCR (ypT0/is) rates. bpCR, breast pathological complete response; HR, hormone receptor; HER2, human epidermal growth factor receptor‐2; IHC, immunohistochemistry; TP, trastuzumab, pertuzumab; tpCR, total pathological complete response; TPPy, trastuzumab, pertuzumab, and pyrotinib.

A total of 36 patients (65.5%, 95% confidence interval [CI]: 51.4%–77.8%) in the TPPy group and 32 patients (60.4%, 95% CI: 46.0%–73.5%) in the TP group achieved total pathological complete response (tpCR, ypT0/isN0) (*p* = 0.585; Figure 2). In the HR‐negative subgroup, a notable but not statistically significant increase in tpCR was observed with the addition of pyrotinib (81.8% vs. 71.9%; *p* = 0.341). Among patients with HER2 immunohistochemistry (IHC) 2+ status, the tpCR rate was 46.7% (7/15) in the TPPy group, which tended to be higher than that in the control group (14.3% [2/14]; *p* = 0.060, Figure 3A). The rates of breast pathological complete response (bpCR, ypT0/is) were 70.9% (95% CI: 57.1%–82.4%) and 64.2% (95% CI: 49.8%–76.9%) in the TPPy and TP groups, respectively (*p* = 0.453, Figure 2). During neoadjuvant chemotherapy, 31 patients (56.4%) in the TPPy group and 23 patients (43.4%) in the TP group achieved clinical complete remission (Table S2). No patients were evaluated as having progressive disease. Two patients in the control arm were not candidates for surgery at the end of four cycles of study treatment and therefore received additional cycles of neoadjuvant treatment. The objective response rates (ORRs) were 92.7% (95% CI: 82.4%–98.0%) and 92.5% (95% CI: 81.8%–97.9%) in the TPPy and TP groups, respectively (*p* = 0.381).

With a median follow‐up of 34.2 months, five recurrences and no deaths were recorded. There were four and one recurrences in the TPPy and TP groups, respectively (*p* = 0.169, Figure S2).

### 2.3 Safety

All patients in the TPPy group and 98.1% of those in the TP group experienced treatment‐related adverse events (TRAEs) of any grade. A total of 40 patients (72.7%) in the TPPy group and 11patients (20.8%) in the TP group experienced grade ≥ 3 TRAEs (Table 2). Despite primary prophylaxis with loperamide and montmorillonite powder in all patients in receiving TPPy, the proportion of patients with grade ≥ 3 diarrhea was 58.1%, which was higher than that in the TP group (0%). Other grade ≥ 3 TRAEs included neutropenia (23.6% in the TPPy group and 15.1% in the TP group) and leukopenia (16.4% and 7.5%, respectively). Serious adverse events (SAEs) occurred in two (3.6%) patients in the TPPy group: one patient developed febrile neutropenia, and the other experienced severe hepatic dysfunction. No major cardiac toxicities were observed. No deaths occurred during neoadjuvant treatment.

**TABLE 2:** Treatment‐related adverse events.

	TPPy group (*N* = 55)		TP group (*N* = 53)	
Adverse events, *N* (%)	Any grade	Grade ≥ 3	Any grade	Grade ≥ 3
Diarrhea	54 (98.2)	32 (58.1)	19 (35.8)	0
Vomiting	42 (76.4)	2 (3.6)	10 (18.9)	0
Anorexia	32 (58.2)	0	11 (20.8)	0
Fatigue	30 (54.5)	0	18 (34)	0
Nausea	24 (43.6)	0	13 (24.5)	0
Alopecia	26 (47.3)	0	29 (54.7)	0
Hypokalemia	20 (36.4)	2 (3.6)	2 (3.8)	0
Peripheral neuropathy	16 (29.1)	0	16 (30.2)	0
Rash	15 (27.3)	0	18 (34)	0
Oral mucositis	12 (21.8)	0	9 (16.9)	0
Infusion reaction	6 (10.9)	0	7 (13.2)	0
Upper respiratory tract infection	1 (1.8)	0	4 (7.5)	0
Leukopenia	29 (52.7)	9 (16.4)	29 (54.7)	4 (7.5)
Neutropenia	28 (50.9)	13 (23.6)	29 (54.7)	8 (15.1)
Anemia	26 (47.3)	2 (3.6)	19 (35.8)	0
Elevated AST	14 (25.5)	1 (1.8)	14 (26.4)	1 (1.9)
Elevated ALT	16 (29.1)	0	15 (28.3)	2 (3.8)

Abbreviations: ALT, alanine aminotransferase; AST, aspartate aminotransferase; TP, trastuzumab, pertuzumab; TPPy, trastuzumab, pertuzumab, and pyrotinib.

In the TPPy group, 52 (94.5%), 31 (56.4%), and 23 (41.8%) patients experienced dose interruptions, dose reductions, and discontinuations of any study drugs, respectively (Table 3). All discontinuations were related to pyrotinib; 47 patients (85.5%) had pyrotinib interruptions, and 31 patients (56.4%) underwent dose reduction of pyrotinib due to intolerable diarrhea. The median relative dose intensity of pyrotinib was 42.0% (Table 4), defined as the ratio of the actual cumulative dose to the planned total dose. Exploratory analysis of the association between dose exposure (categorized into quartiles) and pCR rate indicated that dose intensity of pyrotinib was not significantly correlated with efficacy (Table S3). The median duration of pyrotinib treatment was 77 days (interquartile range [IQR], 48–55) in the experimental arm (Table 4). Moreover, the duration of pyrotinib treatment was also not significantly associated with tpCR rate (Table S3).

**TABLE 3:** Dose modifications of the study drugs.

Events, *N* (%)	TPPy group (*N* = 55)	TP group (*N* = 53)
Treatment‐related AEs of any grade	55 (100.0)	52 (98.1)
Treatment‐related AEs of grade ≥ 3	40 (72.7)	11 (20.8)
Treatment‐related SAEs	2 (3.6)	0
Deaths	0	0
Treatment‐related adverse events		
Any drugs		
Dose interruption	52 (94.5)	6 (11.3)
Dose reduction	31 (56.4)	0
Dose discontinuation	23 (41.8)	0
Pyrotinib		
Dose interruption	47 (85.5)	—
Dose reduction	31 (56.4)	—
Dose discontinuation	23 (41.8)	—

Abbreviations: AEs, adverse events; SAEs, serious adverse events; TP, trastuzumab, pertuzumab; TPPy, trastuzumab, pertuzumab, and pyrotinib.

**TABLE 4:** Pyrotinib dosage exposure.

	TPPy group (*N* = 55)
Actual cumulative exposure dose, mg, median, (IQR)	11,280.0 (5680.0–20,160.0)
Planned exposure dose, mg	26,880.0
Relative actual dose intensity^a^, %, median, (IQR)	42.0 (21.1–75.0)
Relative actual dose intensity, %, mean, (SD)	48.2 (31.1)
Duration on treatment, days, median, (IQR)	77.0 (48.0–55.0)

a* Relative actual dose intensity is defined as the actual cumulative exposure dose/planned exposure dose.

Abbreviations: IQR, interquartile range; SD, standard deviation; TPPy, trastuzumab, pertuzumab, and pyrotinib.

### 2.4 PAM50 Intrinsic Subtypes and tpCR Rate

**FIGURE 4:**
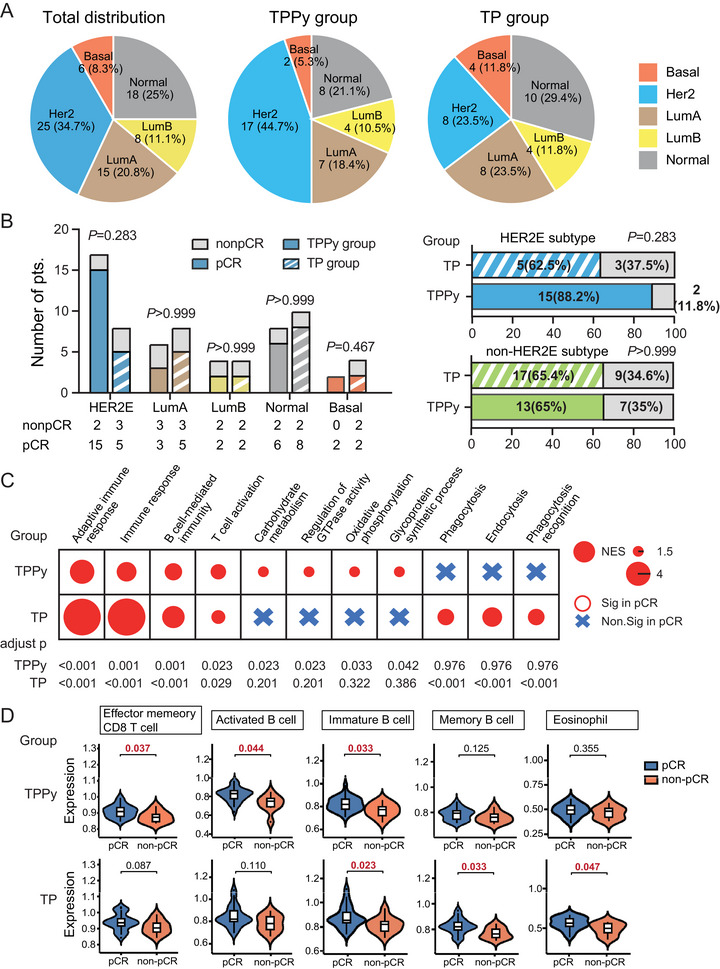
Intrinsic subtypes and enriched pathways predicted response. (A) Pie charts demonstrating distributions of PAM50 intrinsic subtypes in total population, TPPy group and TP group. (B) Bar plots showing pathological responses among five intrinsic subtypes in different treatment groups. *p* values were conducted by chi‐square test and not adjusted. (C) GSEA analyses based on DEGs reflecting pathways commonly or exclusively enriched in different treatment groups. NES was visualized as radium of circles. Pathways with adjusted *p* value < 0.5 and FDR *q* < 0.25 were identified as significant and presented with red circle, while non‐significant presented with blue cross. NES and adjusted *p* values are listed in Table S7. (D) Violin plot showing immune cells predictive for pathological response in TPPy group and TP group separately. *p* values were conducted by logistic test and not adjusted. DEGs, differentially expressed genes; FDR, false discovery rate; GSEA, gene set enrichment analysis; NES, normalized enrichment score; TP, trastuzumab, pertuzumab; TPPy, trastuzumab, pertuzumab, and pyrotinib.

Core needle biopsy samples were available from 90 patients. Total RNA was then successfully extracted from 82 samples and RNA sequencing (RNA‐seq) was successfully performed in 72 samples, among which 71 patients underwent surgery after completing treatment. PAM50 intrinsic subtypes were predicted using bulk RNA‐seq of tumor samples, and the association between intrinsic subtypes and tpCR rates was analyzed (Figure 4A). As shown in Figure 4B, 88.2% (15/17) of patients with HER2‐enriched tumors achieved tpCR after triple HER2 blockade, whereas 62.5% (5/8) of those with HER2‐enriched tumors achieved tpCR after dual TP anti‐HER2 therapy (unadjusted *p* = 0.283). Among patients with non‐HER2‐enriched disease, tpCR rates were 65.0% and 65.4% in the TPPy and TP groups, respectively (unadjusted *p* > 0.999).

### 2.5 Activated Pathways, Immune Cells Infiltration, and tpCR Rate

To identify biomarkers and pathways associated with therapeutic response, differentially expressed genes (DEGs) related to tpCR were identified and subjected to gene set enrichment analysis (GSEA) in the overall population, TPPy group, and TP group (Figure 4C, Figure S3A). HER2‐positive tumors exhibiting adaptive immune response (normalized enrichment score [NES] = 3.79 in TPPy; NES = 5.74 in TP), immune response (NES = 3.03 in TPPy; NES = 5.81 in TP), B‐cell‐mediated immunity (NES = 2.68 in TPPy; NES = 3.50 in TP), and T‐cell activation pathways (NES = 2.43 in TPPy; NES = 2.14 in TP) were significantly associated with higher tpCR rates regardless of TPPy or TP therapy (Figure 4C). Interestingly, pathways related to carbohydrate metabolism (NES = 1.70), regulation of GTPase activity (NES = 1.65), oxidative phosphorylation (NES = 1.58), and glycoprotein synthetic process (NES = 1.61) were exclusively correlated with tpCR in the TPPy group, suggesting that tumors with upregulated biological process of oxidative phosphorylation were more likely to respond to pyrotinib. Conversely, pathways associated with phagocytosis (NES = 2.57), endocytosis (NES = 3.01), and phagocytosis recognition (NES = 2.57) were specifically predictive of tpCR in the TP group, possibly due to the mechanism of action of monoclonal antibodies used in this regimen (Figure 4C). Furthermore, Kyoto Encyclopedia of Genes and Genomes (KEGG) pathway analysis demonstrated that linoleic acid metabolism and ether lipid metabolism pathways were uniquely associated with tpCR in the TPPy group, whereas Toll‐like receptor signaling, FcεRI signaling, and regulation of autophagy pathways were associated with tpCR in the TP group (Figure S3A).

GSEA results indicated that immune activation was associated with a higher likelihood of tpCR in both triple and dual HER2 blockade therapies (Figure S3B). Specific immune cell subsets associated with tpCR were further investigated using single sample gene set enrichment analysis (ssGSEA). As shown in Figure 4D, HER2‐positive tumors with a higher infiltration level of effector memory CD8+ T cell (unadjusted *p* = 0.03) and activated B cell (unadjusted *p* = 0.044) were more likely to respond to TPPy treatment. In contrast, in the TP group, tpCR was significantly associated with higher levels of memory B cell (unadjusted *p* = 0.033) and eosinophil cell (unadjusted *p* = 0.047). Notably, immature B‐cell infiltration was correlated with tpCR in both treatment groups (unadjusted *p* = 0.033 for the TPPy group; unadjusted *p* = 0.023 for the TP group).

## 3 Discussion

Our study demonstrated that triple HER2 blockade with TPPy achieved a tpCR rate comparable to that of TP in the neoadjuvant treatment of HER2‐positive breast cancer. To our knowledge, this is the first randomized trial to evaluate triple HER2 blockade versus dual anti‐HER2 therapy in the neoadjuvant setting. The study was prematurely terminated based on a planned futility analysis. Moreover, a higher incidence of severe diarrhea events was observed in the triple HER2 blockade treatment group.

The combination of pyrotinib with neoadjuvant chemotherapy and trastuzumab in HER2‐positive breast cancer has been investigated in several clinical trials as summarized in Table S4, with tpCR rates ranging from 41.0% to 73.7% [8, 10, 11, 12, 13]. This regimen was first examined in a small pilot study, in which 19 patients received four cycles of epirubicin, cyclophosphamide, and pyrotinib followed by four cycles of docetaxel, trastuzumab, and pyrotinib before surgery, achieving a tpCR rate of 73.7% [14]. A subsequent multicenter single‐arm trial using the same regimen in 175 patients with stage II–III HER2‐positive breast cancer reported a tpCR rate of 68.6% [10]. Furthermore, the phase III randomized PHEDRA trial demonstrated that adding pyrotinb to trastuzumab and docetaxel significantly improved tpCR [8]. These findings suggested that combining chemotherapy and trastuzumab with pyrotinib could represent a promising neoadjuvant approach. However, the addition of pyrotinib to chemotherapy plus dual HER2‐blockade with trastuzumab and pertuzumab has not been previously investigated. Therefore, we designed the current study to address this gap.

Although adding pyrotinib to nab‐paclitaxel and TP did not increase the tpCR rate, the notably high efficacy observed in the control group suggests that four cycles of nab‐paclitaxel monotherapy may represent an optimal chemotherapy backbone for dual HER2 blockade with TP. Nab‐paclitaxel is an albumin‐bound particle formulation of paclitaxel, devoid of any solvent excipients. It offers several potential advantages over solvent‐based taxanes, including the ability to deliver higher doses, shorter infusion times, and increased drug concentrations. In preclinical mouse xenograft models, nab‐paclitaxel demonstrated increased intratumoral concentrations and greater binding to and transport across endothelial cells compared with paclitaxel [15]. In metastatic breast cancer, the CA024 study first demonstrated that nab‐paclitaxel significantly improved the ORR and progression‐free survival compared with conventional paclitaxel as first‐line treatment [16]. In the neoadjuvant setting, nab‐paclitaxel combined with targeted therapy also showed a notably high efficacy. The GeparSepto trial demonstrated a significant increase in the tpCR rate and improved outcomes with nab‐paclitaxel compared to solvent‐based paclitaxel in early breast cancer patients [17, 18]. The HELEN‐006 study reported that six cycles of nab‐paclitaxel plus TP achieved a tpCR rate of 66.3% in neoadjuvant treatment of HER2‐positive breast cancer [19]. In our study, four cycles of nab‐paclitaxel plus TP yielded a tpCR rate of 60.4%. However, since this was a phase II trial with a small sample size, the high tpCR rate requires confirmation in larger cohorts.

The negative result in our study may be explained by a “ceiling effect” of anti‐HER2 neoadjuvant therapy, as evidenced by the notably high efficacy observed in the control group. With the recent development of HER2‐directed antibody‐drug conjugates, single‐agent SHR‐A1811 neoadjuvant therapy achieved a pCR rate of 63.2%, which was comparable to that of six cycles of nab‐paclitaxel combined with carboplatin and TP regimen (pCR rate 64.4%). However, adding pyrotinib to SHR‐A1811 did not further increase the pCR rate (62.5%) in the neoadjuvant treatment of HER2‐positive breast cancer, again suggesting a potential “ceiling effect” [20].

HER2‐positive breast cancer is a biologically heterogeneous disease. Tumors with positive versus negative HR status display distinct molecular characteristics that influence treatment response. Moreover, HER2‐positive breast cancer comprises diverse intrinsic subtypes—including HER2‐enriched, basal‐like, Luminal A, and Luminal B—that demonstrate varying degrees of sensitivity to anti‐HER2 therapy [21]. Our study showed that the triple HER2 blockade plus chemotherapy achieved an 81.8% tpCR rate in the HR–/HER2+ tumors, consistent with prior studies of dual‐targeted anti‐HER2 neoadjuvant therapy [4, 22, 23], likely due to the dominant HER2‐driven biological process in this subgroup [21, 24]. Furthermore, transcriptomic RNA‐seq analysis revealed that HER2‐enriched tumors exhibited a tpCR rate of 88.2% in the TPPy group, suggesting that triple HER2 blockade may further enhance treatment response in this molecular subtype.

An interesting finding in our study was that the tpCR rate among patients with HER2 IHC 2+/FISH‐positive tumors in the TP group was only 14.3%, whereas the rate tended to be higher, reaching 46.7%, in the TPPy group. A lower tpCR rate in HER2 IHC 2+/FISH‐positive patients compared with HER2 IHC 3+ patients has been reported in previous studies [25, 26, 27]. However, the benefit of pyrotinib in HER2 2+ patients has not been consistently observed [10, 20]. In our study, patients with HER2 IHC 2+ tumors had a higher proportion of ER‐positive disease (Table S5). Regarding PAM50 subtypes (Figure S4), only 14.3% (3 out of 21) IHC 2+ tumors were classified as the HER2‐enriched subtype, significantly lower than 43.1% among IHC 3+ tumors (22 out of 51) (unadjusted *p* = 0.019). Differences in intrinsic subtype may contribute to the differential benefit of pyrotinib. A more comprehensive investigation of the underlying biological mechanisms is warranted to identify more accurate predictive biomarkers.

Regarding transcriptomic features, in our study, it was found that tumors exhibiting higher activity—metabolic carbohydrate metabolism, glycoprotein synthetic process, and oxidative phosphorylation—were more likely to benefit from the combination of pyrotinib and dual HER2 blockade. Notably, regarding tumor immune microenvironment, B‐cell clusters, including activated, immature, and memory B cell, were significantly associated with pCR. This finding is consistent with prior reports indicating that B‐cell immune signatures are associated with higher pCR rates and improved clinical outcomes in patients with HER2‐positive breast cancer [28, 29, 30].

As for genomic alterations, PIK3CA mutation may be a predictive biomarker for HER2‐targeted therapy. Previous studies have reported that HER2‐positive breast cancers harboring PIK3CA mutations may exhibit reduced sensitivity to trastuzumab, pertuzumab, and lapatinib [31, 32, 33, 34]. However, no consensus has been met for pyrotinib. In advanced setting, co‐mutations of PIK3CA and TP53 have been reported as a predictor for poor pyrotinib response [35]. In a phase II neoadjuvant study, activating PIK3CA mutations were associated with reduced responsiveness to pyrotinib, with a pCR rate of 80.8% in wild‐type versus 26.3% in mutated tumors [36]. Conversely, in the single‐armed phase II NeoATP trial, in which four cycles of trastuzumab, pyrotinib, and paclitaxel‐cisplatin chemotherapy were offered in stage II‐III HER2 positive breast cancer patients in neoadjuvant setting, the pCR rate was comparable in patients with or without PIK3CA mutations (69.2% vs. 70.0%).The authors interpreted these findings to suggest that pyrotinib as a TKI may reverse the HER2‐targeting resistance caused by PIK3CA mutations [11]. In our study, GSEA of the PI3K/AKT/mTOR signaling pathways was performed to evaluated the potential role of PIK3CA mutation for treatment efficacy in both TPPy and TP groups. However, no significant differences were observed in GO‐PI3K signaling, GO‐regulation of PI3K signaling, or KEGG‐mTOR signaling between responders and non‐responders in either group (all adjusted *p* > 0.05; Table S6). Therefore, in our cohort, PI3K/AKT/mTOR pathways failed to predict the efficacy of triple or dual HER2‐targeted regimens. Nevertheless, the PAM signaling analyses could not reflect the status of PIK3CA precisely. Thus, combined analyses of transcriptomic and whole exome sequencing are warranted to elucidate the precise biological mechanisms underlying the relationship between PIK3CA mutation and pyrotinib response.

Currently, there is no published safety data on triple HER2 blockade in HER2‐positive breast cancer patients. In our study, the most common adverse event in the TPPy group was grade 3 diarrhea, occurring in 58.1% of patients despite primary prophylactic antidiarrhea medication. EGFR and HER2 are both expressed on intestinal epithelial cell membranes and act in concert to negatively regulate chloride secretion via the PI3K and protein kinase C pathways. Pertuzumab inhibits the HER2 receptor at the extracellular domain II region to promote HER2 signaling and downstream PI3K signaling inhibition, which may increase the rate of diarrhea when combined with trastuzumab therapy [37]. The PEONY trial demonstrated that the combination of TP with docetaxel resulted in a higher incidence of diarrhea of any grade (38.5%) compared with trastuzumab plus docetaxel (16.4%), a finding also observed in the adjuvant APHINITY trial, particularly among elderly patients [5, 38]. TKI‐induced diarrhea is common, and its mechanisms are multifactorial, involving inhibition of intestinal epithelial cell proliferation, excessive chloride ion secretion, inflammation, bile acid malabsorption, and dysbiosis of the intestinal microbiota [39, 40]. The PHEDRA trial reported a 44.4% incidence of grade 3 diarrhea with the combination of trastuzumab and pyrotinib [8]. In our study, triple HER2 blockade combined with nab‐paclitaxel led to a high rate of severe diarrhea, possibly due to more complete HER2 pathway inhibition and stronger EGFR blockade. Although dose intensity did not significantly influence the tpCR rate, the limited sample size in this phase II trial may have affected the precision of these findings. Better treatment adherence may potentially improve therapeutic efficacy. The phase II CONTROL trial investigated antidiarrheal prophylaxis and neratinib dose‐escalation strategies for diarrhea prevention; both approaches improved the tolerability of neratinib [41]. Future studies evaluating triple HER2 blockade should incorporate optimized diarrhea management strategies—such as gradual dose escalation combined with antidiarrheal medications—which may enhance pyrotinib tolerability and, in turn, influence treatment outcomes.

There were several limitations in our study. First, the preplanned futility interim monitoring analysis led to the early study termination, as the conditional power was lower than the planned threshold, which have resulted in insufficient power for efficacy evaluation. Second, a higher rate of treatment discontinuation was observed in the TPPy group, primarily due to poor tolerance, which could have affected treatment adherence. Third, the median follow‐up duration of 34.2 months may be inadequate for definitive outcome assessment in HER2‐positive breast cancer. Longer follow up is required to determine whether pyrotinib exerts a beneficial effect on long‐term survival. Fourth, for the transcriptomic analysis of biological pathways, multivariate statistical testing could not be performed because of the limited number of tumor samples (*N* = 72). Therefore, the key pathways and intrinsic features identified in this study should be regarded as exploratory findings and require validation in larger patient cohorts. Finally, the tpCR rate in the control TP group was much higher than expected, and this “ceiling effect” poses additional challenges in further improving tpCR with pyrotinib in the neoadjuvant treatment of HER2‐positive breast cancer.

## 4 Conclusion

Triple HER2 blockade combined with nab‐paclitaxel did not significantly improve the tpCR rate compared with dual TP therapy in the neoadjuvant treatment of HER2‐positive breast cancer and was associated with a high incidence of severe diarrhea. Because the study was terminated early and was underpowered relative to its original objective, further validation in larger cohorts is warranted before definitive conclusions can be drawn. Additional biomarker analyses to optimize patient selection may help guide future clinical trials.

## 5 Materials and Methods

### 5.1 Study Design

This multicenter, randomized, open‐label, phase II study was conducted to evaluate the efficacy and safety of triple versus dual anti‐HER2 therapy in the neoadjuvant treatment of HER2‐positive breast cancer (ClinicalTrials.gov identifier: NCT04398914). Eligible patients were 18–75 years of age with histologically confirmed stage II–III invasive HER2‐positive breast cancer (primary tumor >2 cm in diameter) and were recruited from Shanghai Ruijin Hospital and Zhejiang Shangyu People's Hospital in China. HER2 positivity was defined as 3+ by IHC or as HER2 gene amplification confirmed by in situ hybridization (ISH). Key inclusion criteria included an Eastern Cooperative Oncology Group (ECOG) performance status of 0 or 1, known ER and progesterone receptor (PR) status, and a baseline left ventricular ejection fraction ≥ 50% as assessed by echocardiography. Exclusion criteria comprised patients with metastatic disease (stage IV); a history of other malignancies, except adequately treated cervical carcinoma in situ, nonmelanoma skin cancer, stage I uterine cancer, or papillary thyroid microcarcinoma; previous anticancer therapy or radiotherapy for any malignancy; or clinically significant cardiovascular disease. The study protocol was approved by the Ethics Committee at each participating center and conducted in accordance with the Declaration of Helsinki and Good Clinical Practice guidelines. Written informed consent was obtained from all participants prior to study enrollment.

### 5.2 Randomization and Masking

Patients were randomly assigned (1:1) to receive either TP plus nab‐paclitaxel (TP group) or the same regimen combined with pyrotinib (TPPy group). Randomization was performed using a stratified, permuted block design with a block size of four. Stratification factors included disease stage (stage II vs. stage III) and HR status (positive vs. negative). This was an open‐label trial; therefore, investigators, patients, and statisticians were not blinded to treatment allocation.

### 5.3 Procedure

Enrolled patients received intravenous trastuzumab (8 mg/kg loading dose on day 1 in cycle 1, followed by 6 mg/kg maintenance dose on day 1 in cycles 2–4) combined with intravenous pertuzumab (840 mg loading dose on day 1 in cycle 1, followed by 420 mg maintenance doses on day 1 in cycles 2–4) and nab‐paclitaxel (100 mg/m^2^ on days 1, 8 and 15 of each 21‐day cycle) for four cycles as neoadjuvant treatment. Patients in the TPPy group additionally received oral pyrotinib at a dose of 320 mg once daily. The clinical response was determined by comparing the largest tumor diameter at baseline, on day 1 of cycles 2–4 during therapy, and before surgery, as assessed by ultrasonography and/or magnetic resonance imaging (whenever preferred) according to Response Evaluation Criteria in Solid Tumors (RECIST) version 1.1 [42]. Surgery was performed within 3 weeks after the final dose of neoadjuvant chemotherapy for all eligible patients. After surgery, adjuvant treatments—including chemotherapy, radiotherapy, and anti‐HER2–targeted therapy—were administered according to clinical practice guidelines. Patients who discontinued neoadjuvant therapy due to toxicity, exhibited progressive disease, or were deemed ineligible for surgery received subsequent anticancer therapy at the physician's discretion following local treatment standards.

Loperamide plus montmorillonite powder was recommended as primary prophylaxis for diarrhea during pyrotinib treatment. Temporary interruption and dose reduction of pyrotinib were implemented for diarrhea management. For the TP regimen, dose adjustment was not permitted unless body weight changes occurred; however, treatment could be interrupted or discontinued due to toxicity—particularly cardiotoxicity. If treatment suspension exceeded 6 weeks, dose modification was permanently discontinued. For nab‐paclitaxel, dose adjustment, interruption, and discontinuation were permitted. Gradual dose reduction was allowed, but patients were withdrawn from study treatment if further reduction was required. Once the dose was reduced, re‐escalation was not permitted. Nab‐paclitaxel treatment was permanently discontinued when suspension exceeded 3 weeks.

The final safety assessment was conducted approximately 28 days after the last dose of neoadjuvant therapy. Patients were followed up for survival every 3 months (±28 days) during the first year and every 6 months thereafter until disease progression, recurrence, or up to 5 years after randomization of the last enrolled patient, whichever occurred first.

### 5.4 Outcomes

The primary endpoint was the tpCR, evaluated by investigators and defined as the absence of residual invasive cancer in both the breast and axillary lymph nodes (ypT0/isN0). Secondary endpoints included the bpCR (defined as ypT0/is) rate, the ORR (defined as the proportion of patients who achieved a best overall response of complete or partial response during neoadjuvant therapy), survival outcomes, and safety.

### 5.5 Sample Obtain and RNA‐seq

Treatment‐naïve tumor samples were obtained in two ways. Fresh tumor samples were acquired during core needle biopsy and conserved in RNAlater stabilization reagent (Thermo Fisher Scientific, USA). For patients without fresh sample, formalin‐fixed paraffin‐embedded (FFPE) blocks were obtained, and RNA was extracted by RNeasy FFPE Kit (Qiagen, Beverly, USA).

Strand‐specific libraries were constructed using the Stranded mRNA‐seq (NR602) (Vazyme, Nanjing, China) for frozen samples and Watchmaker RNA Library prep kits with polaris depletion for FFPE samples. RNA‐seq was carried out by 2× 150 bp paired‐end using the Illumina NovaSeq X Plus instrument. The raw data were processed by Skewer, and quality was examined with FastQC (v0.11.5). Clean reads were mapped to the human genome hg38 using STAR. The original sequence reads of the known genes were counted using StringTie (version 2.2.1).

### 5.6 Transcriptome Analysis and Pathway Prediction

Gene expression levels in RNA‐seq sequencing were estimated as fragments per kilobase million (FPKM) values. To remove the variance caused by different library construction, batch effects were removed by Combat function via sva (v3.54.0) and tested by principal component analysis (PCA) via prcomp (v3.6.2).

PAM50 intrinsic molecular subtypes was classified by genefu (v2.37.0) and its distribution was calculated in whole population and subgroups separately. Immune cell infiltration was predicted by ssGSEA algorithm via gsva (v2.0.5) and their correlations with pCR were analyzed both in the TP and TPPy groups by Wilcoxon test. Significant pathways predictive for treatment efficacy were analyzed via gsva (v2.0.5), especially in HALLMARK and KEGG pathways. Furthermore, GSEA analyses were performed and pathway enrichment scores were calculated by msigdbr (v10.0.1) and clusterProfiler (v4.14.6) so that different biological processes crucial for pCR were compared among whole population, pyrotinib subgroup, and control subgroup. Significance of GSEA pathway was defined as false discovery rate (FDR) *q* value < 0.25 and adjusted *p* value < 0.05.

### 5.7 Statistical Analysis

The study was designed to achieve an 80% power to detect a 15% increase in the tpCR rate with pyrotinib therapy, at a one‐sided α level of 0.1. The expected tpCR rate for four cycles of nab‐paclitaxel plus TP was assumed to be 40% based on data from the NeoSphere and PEONY trials [4, 5]. An absolute increase of 15% in tpCR was hypothesized with the addition of pyrotinib [43]. A total of 216 patients were planned for enrollment, accounting for an estimated 10% of patients who might be unevaluable for pathological response assessment. An interim futility analysis was planned after enrollment of half the target sample size (108 patients). Conditional power was calculated via simulation based on the interim tpCR results; if the probability of achieving a positive result was <50%, early trial termination was recommended. By July 2023, a total of 108 patients had been enrolled and analyzed for tpCR. A noninformative beta (1,1) prior was applied to derive the posterior distribution and corresponding probability of superiority. Post hoc Bayesian analysis indicated a 7.0% predictive probability that the experimental arm would be superior to the control arm if full enrollment were completed (Figure S1). Furthermore, a higher incidence of severe gastrointestinal adverse events—particularly severe diarrhea—and a high early discontinuation rate of pyrotinib were observed in the triple HER2 blockade group, leading investigators to terminate enrollment.

Efficacy analyses were performed on the full analysis set, which included all randomized patients who received at least one dose of the study medication, with analyses conducted according to their original randomization assignments. Safety analyses included all patients who received at least one dose of study treatment based on the actual treatment received. The tpCR rates between the two groups were compared using a two‐sided *χ* ^2^ test, stratified by HR status and tumor stage. Results from Fisher's exact test were also reported. All statistical analyses were conducted using SPSS software (version 22.0). Sample size estimation was performed using the “Superiority by a Margin Test for the Difference Between Two Proportions” procedure in PASS software (version 15.0).

## Author Contributions

All authors had full access to the data and take responsibility for the integrity and accuracy of the analyses. K.W.S. contributed to the conceptualization and design of the trial. X.S.C. and J.H.H. were responsible for data collection and assembly. J.H.H., H.Y.W., Y.W.T., and H.T.S. performed the statistical analyses and drafted the manuscript. W.L.R., J.Y., W.Q.G., S.J.Z., J.H., J.Y.W., O.H., J.R.H., W.G.C., and Y.F.L. contributed to patient enrollment. All authors participated in manuscript preparation and approved the final version for submission.

## Funding

This work was supported by grants from the National Natural Science Foundation of China (grant numbers 82072937, 82072897, 82403868, and 82573726), the Natural Science Foundation of the Shanghai Science and Technology Committee (grant number 23ZR1439500), the National Research Center for Translational Medicine at Shanghai (NRCTM(SH)‐2023‐14), the Shanghai Municipal Health Commission Clinical Research Project (grant number 20214Y0189), the Innovative Research Team of High‐Level Local Universities in Shanghai (SHSMU‐ZDCX20212200), and the Science and Technology Commission of Shanghai Municipality Shanghai Sailing Program (22YF1426500). These funding sources had no role in the design and conduct of the study; the collection, management, analysis, and interpretation of the data; the preparation, review, or approval of the manuscript; or the decision to submit the manuscript for publication.

## Ethics Statement

The study was approved by the institutional ethics committees of Ruijin Hospital and Shangyu People's Hospital (approval number: 2020 Clinical Ethics Review [76‐3]). Written informed consent was obtained from all participants in accordance with the principles of the Declaration of Helsinki. This trial was registered at ClinicalTrials.gov (identifier: NCT04398914).

## Conflicts of Interest

Jing Li is an employee in Jiangsu Hengrui Pharmaceuticals Co., Ltd. The study drugs, pyrotinib and nab‐paclitaxel, were provided by Jiangsu Hengrui Pharmaceuticals Co., Ltd. The remaining authors declare no conflicts of interest.

## Supporting information



Table S1 Adjuvant treatment in study patients.
S2 Clinical response in the TPPy and TP groups.
Table S3 tpCR rate in patients in the TPPy group according to relative dose intensity quantile and median days on treatment.
Table S4 Clinical studies on neoadjuvant pyrotinib.
Table S5 Patient characteristics according to HER2 IHC scores.
Table S6 PI3K/AKT/mTOR pathway analyses related with pCR in TPPy and TP group.
Table S7 GSEA pathway analyses related with pCR in TPPy and TP group.
Figure S1. Bayesian estimation at interim analysis. The tpCR rate was estimated to be 65.8% (95% PI, 50.7 to 68.9%) in TPPy group, and 60.2% (95% PI, 56.4 to 74.0%) in TP group. The predictive probability of superior was 7% and the probability of equivalence was 92.9% in full planned enrollment. TPPy, trastuzumab, pertuzumab and pyrotinib; TP, trastuzumab, pertuzumab; tPCR, total pathological complete remission; PI, predictive interval.
Figure S2. Recurrence‐free survival comparison between treatment arms. TPPy, trastuzumab, pertuzumab and pyrotinib; TP, trastuzumab, pertuzumab.
Figure S3. KEGG pathways and immune cell infiltration correlated with pCR. (A) Heatmap demonstrating KEGG pathways exclusively related with pCR in TPPy group and TP group. Upregulated alpha linoleic acid metabolism (unadjusted P = 0.027), linoleic acid metabolism (unadjusted P = 0.021) and ether lipid metabolism (unadjusted P = 0.043) was correlated with higher likelihood of pCR in TPPy group; On the other hand, enriched pathways of toll‐like receptor signaling (unadjusted P = 0.032), Fcε RI signaling (unadjusted P = 0.046), Regulation of autophagy (unadjusted P = 0.016) and Histidine metabolism (unadjusted P = 0.022) were significantly related with higher pCR rate in TP group. (B) Violin plot showing immune cells predictive for pathological response in total population. TPPy, trastuzumab, pertuzumab and pyrotinib; TP, trastuzumab, pertuzumab; KEGG, Kyoto Encyclopedia of Genes and Genomes.
Figure S4 PAM50 intrinsic subtypes according to HER2 IHC scores. HER2, human epidermal growth factor receptor‐2; IHC, immunohistochemistry.

## Data Availability

The raw clinical data are protected and cannot be shared due to data privacy regulations. De‐identified datasets supporting the findings of this study are available for academic purposes upon reasonable request from the corresponding authors, Kunwei Shen or Xiaosong Chen, for up to 5 years following publication, pending approval by the respective institutional ethics committees.

## References

[1] R. M. Sareyeldin , I. Gupta , I. Al‐Hashimi , et al., “Gene Expression and miRNAs Profiling: Function and Regulation in Human Epidermal Growth Factor Receptor 2 (HER2)‐Positive Breast Cancer,” Cancers 11, no. 5 (2019): 646. 31083383 10.3390/cancers11050646 PMC6562440

[2] L. M. Spring , G. Fell , A. Arfe , et al., “Pathologic Complete Response After Neoadjuvant Chemotherapy and Impact on Breast Cancer Recurrence and Survival: A Comprehensive Meta‐Analysis,” Clinical Cancer Research 26, no. 12 (2020): 2838–2848. 32046998 10.1158/1078-0432.CCR-19-3492 PMC7299787

[3] P. Cortazar , L. Zhang , M. Untch , et al., “Pathological Complete Response and Long‐Term Clinical Benefit in Breast Cancer: The CTNeoBC Pooled Analysis,” Lancet 384, no. 9938 (2014): 164–172. 24529560 10.1016/S0140-6736(13)62422-8

[4] L. Gianni , T. Pienkowski , Y. H. Im , et al., “Efficacy and Safety of Neoadjuvant Pertuzumab and Trastuzumab in Women With Locally Advanced, Inflammatory, or Early HER2‐positive Breast Cancer (NeoSphere): A Randomised Multicentre, Open‐Label, Phase 2 Trial,” Lancet Oncology 13, no. 1 (2012): 25–32. 22153890 10.1016/S1470-2045(11)70336-9

[5] Z. Shao , D. Pang , H. Yang , et al., “Efficacy, Safety, and Tolerability of Pertuzumab, Trastuzumab, and Docetaxel for Patients with Early or Locally Advanced ERBB2‐Positive Breast Cancer in Asia: The PEONY Phase 3 Randomized Clinical Trial,” JAMA Oncology 6, no. 3 (2020): e193692. 31647503 10.1001/jamaoncol.2019.3692 PMC6813591

[6] L. Xu , Y. Xie , Q. Gou , et al., “HER2‐Targeted Therapies for HER2‐Positive Early‐Stage Breast Cancer: Present and Future,” Frontiers in Pharmacology 15 (2024): 1446414. 39351085 10.3389/fphar.2024.1446414 PMC11439691

[7] X. Li , C. Yang , H. Wan , et al., “Discovery and Development of Pyrotinib: A Novel Irreversible EGFR/HER2 Dual Tyrosine Kinase Inhibitor With Favorable Safety Profiles for the Treatment of Breast Cancer,” European Journal of Pharmaceutical Sciences 110 (2017): 51–61. 28115222 10.1016/j.ejps.2017.01.021

[8] J. Wu , Z. Jiang , Z. Liu , et al., “Neoadjuvant Pyrotinib, Trastuzumab, and Docetaxel for HER2‐Positive Breast Cancer (PHEDRA): A Double‐Blind, Randomized Phase 3 Trial,” BMC Medicine 20, no. 1 (2022): 498. 36575513 10.1186/s12916-022-02708-3 PMC9795751

[9] A. Canonici , L. Ivers , N. T. Conlon , et al., “HER‐Targeted Tyrosine Kinase Inhibitors Enhance Response to Trastuzumab and Pertuzumab in HER2‐Positive Breast Cancer,” Investigational New Drugs 37, no. 3 (2019): 441–451. 30062574 10.1007/s10637-018-0649-y

[10] Q. Shi , X. Qi , P. Tang , et al., “A Multicenter Single‐Arm Trial of Neoadjuvant Pyrotinib and Trastuzumab Plus Chemotherapy for HER2‐Positive Breast Cancer,” Medicine Communications 4, no. 6 (2023): e435. 38077249 10.1002/mco2.435 PMC10701463

[11] W. Yin , Y. Wang , Z. Wu , et al., “Neoadjuvant Trastuzumab and Pyrotinib for Locally Advanced HER2‐Positive Breast Cancer (NeoATP): Primary Analysis of a Phase II Study,” Clinical Cancer Research 28, no. 17 (2022): 3677–3685. 35713517 10.1158/1078-0432.CCR-22-0446

[12] Z. Liu , C. Wang , X. Chen , et al., “Pathological Response and Predictive Role of Tumour‐Infiltrating Lymphocytes in HER2‐Positive Early Breast Cancer Treated With Neoadjuvant Pyrotinib Plus Trastuzumab and Chemotherapy (Panphila): A Multicentre Phase 2 Trial,” European Journal of Cancer 165 (2022): 157–168. 35235873 10.1016/j.ejca.2022.01.022

[13] Y. Ding , W. Mo , X. Xie , et al., “Neoadjuvant Pyrotinib Plus Trastuzumab, Docetaxel, and Carboplatin in Early or Locally Advanced Human Epidermal Receptor 2‐Positive Breast Cancer in China: A Multicenter, Randomized, Double‐Blind, Placebo‐Controlled Phase 2 Trial,” Oncology Research and Treatment 46, no. 7–8 (2023): 303–311. 37302393 10.1159/000531492

[14] J. Xuhong , X. Qi , P. Tang , et al., “Neoadjuvant Pyrotinib Plus Trastuzumab and Chemotherapy for Stage I‐III HER2‐Positive Breast Cancer: A Phase II Clinical Trial,” Oncologist 25, no. 12 (2020): e1909–e1920. 33000490 10.1002/onco.13546 PMC8108050

[15] N. Desai , V. Trieu , Z. Yao , et al., “Increased Antitumor Activity, Intratumor Paclitaxel Concentrations, and Endothelial Cell Transport of Cremophor‐Free, Albumin‐Bound Paclitaxel, ABI‐007, Compared With Cremophor‐Based Paclitaxel,” Clinical Cancer Research 12 (2006): 1317–1324. 16489089 10.1158/1078-0432.CCR-05-1634

[16] W. J. Gradishar , D. Krasnojon , S. Cheporov , et al., “Significantly Longer Progression‐Free Survival With Nab‐Paclitaxel Compared With Docetaxel as First‐Line Therapy for Metastatic Breast Cancer,” Journal of Clinical Oncology 27, no. 22 (2009): 3611–3619. 19470941 10.1200/JCO.2008.18.5397

[17] M. Untch , C. Jackisch , A. Schneeweiss , et al., “Nab‐Paclitaxel Versus Solvent‐Based Paclitaxel in Neoadjuvant Chemotherapy for Early Breast Cancer (GeparSepto‐GBG 69): A Randomised, Phase 3 Trial,” Lancet Oncology 17, no. 3 (2016): 345–356. 26869049 10.1016/S1470-2045(15)00542-2

[18] M. Untch , C. Jackisch , A. Schneeweiss , et al., “NAB‐Paclitaxel Improves Disease‐Free Survival in Early Breast Cancer: GBG 69‐GeparSepto,” Journal of Clinical Oncology 37, no. 25 (2019): 2226–2234. 31082269 10.1200/JCO.18.01842

[19] X. C. Chen , D. C. Jiao , J. H. Qiao , et al., “De‐Escalated Neoadjuvant Weekly Nab‐Paclitaxel With Trastuzumab and Pertuzumab Versus Docetaxel, Carboplatin, Trastuzumab, and Pertuzumab in Patients With HER2‐Positive Early Breast Cancer (HELEN‐006): A Multicentre, Randomised, Phase 3 Trial,” Lancet Oncology 26, no. 1 (2025): 27–36. 39612919 10.1016/S1470-2045(24)00581-3

[20] J. J. Li , Z. H. Wang , L. Chen , et al., “Efficacy and Safety of Neoadjuvant SHR‐A1811 With or Without Pyrotinib in Women With Locally Advanced or Early HER2‐Positive Breast Cancer: A Randomized, Open‐Label, Phase II Trial,” Annals of Oncology 36, no. 6 (2025): 651–659. 40049447 10.1016/j.annonc.2025.02.011

[21] A. G. Waks , O. Martínez‐Sáez , P. Tarantino , et al., “Dual HER2 Inhibition: Mechanisms of Synergy, Patient Selection, and Resistance,” Nature Reviews Clinical Oncology 21, no. 11 (2024): 818–832. 10.1038/s41571-024-00939-2 39271787

[22] J. Baselga , I. Bradbury , H. Eidtmann , et al., “Lapatinib With Trastuzumab for HER2‐Positive Early Breast Cancer (NeoALTTO): A Randomised, Open‐Label, Multicentre, Phase 3 Trial,” Lancet 379, no. 9816 (2012): 633–640. 22257673 10.1016/S0140-6736(11)61847-3 PMC5705192

[23] S. A. Jacobs , A. Robidoux , J. Abraham , et al., “NSABP FB‐7: A Phase II Randomized Neoadjuvant Trial With Paclitaxel + Trastuzumab and/or Neratinib Followed by Chemotherapy and Postoperative Trastuzumab in HER2(+) Breast Cancer,” Breast Cancer Research 21, no. 1 (2019): 133. 31796073 10.1186/s13058-019-1196-y PMC6892191

[24] A. Prat , T. Pascual , C. De Angelis , et al., “HER2‐Enriched Subtype and ERBB2 Expression in HER2‐Positive Breast Cancer Treated With Dual HER2 Blockade,” JNCI: Journal of the National Cancer Institute 112, no. 1 (2020): 46–54. 31037288 10.1093/jnci/djz042 PMC7850037

[25] D. Jiao , G. Li , H. Dai , et al., “Comparison of the Response to Neoadjuvant Therapy Between Immunohistochemistry HER2 (3+) and HER2 (2+)/ISH+ Early‐Stage Breast Cancer: A Retrospective Multicenter Cohort Study,” Oncologist 29, no. 7 (2024): e877–e886. 38537665 10.1093/oncolo/oyae047 PMC11224972

[26] N. Tung , F. Zhao , A. DeMichele , et al., “Predicting Pathologic Complete Response (pCR) From Clinicopathologic Variables and HER2DX Genomic Test in Stage II/III HER2+ Breast Cancer Treated With Taxane, Trastuzumab, and Pertuzumab (THP): Secondary Results From the EA1181/CompassHER2 pCR Trial,” Journal of Clinical Oncology 43, no. supplement S16 (2025): 501–501.

[27] C. Marchiò , L. Annaratone , A. Marques , et al., “Evolving Concepts in HER2 Evaluation in Breast Cancer: Heterogeneity, HER2‐Low Carcinomas and Beyond,” Seminars in Cancer Biology 72 (2021): 123–135. 32112814 10.1016/j.semcancer.2020.02.016

[28] A. Fernandez‐Martinez , T. Pascual , B. Singh , et al., “Prognostic and Predictive Value of Immune‐Related Gene Expression Signatures vs Tumor‐Infiltrating Lymphocytes in Early‐Stage ERBB2/HER2‐Positive Breast Cancer: A Correlative Analysis of the CALGB 40601 and PAMELA Trials,” JAMA Oncology 9, no. 4 (2023): 490–499. 36602784 10.1001/jamaoncol.2022.6288 PMC9857319

[29] M. Rediti , A. Fernandez‐Martinez , D. Venet , et al., “Immunological and Clinicopathological Features Predict HER2‐Positive Breast Cancer Prognosis in the Neoadjuvant NeoALTTO and CALGB 40601 Randomized Trials,” Nature Communications 14, no. 1 (2023): 7053. 10.1038/s41467-023-42635-2 PMC10624889 37923752

[30] A. Fernandez‐Martinez , M. Rediti , G. Tang , et al., “Tumor Intrinsic Subtypes and Gene Expression Signatures in Early‐Stage ERBB2/HER2‐Positive Breast Cancer: A Pooled Analysis of CALGB 40601, NeoALTTO, and NSABP B‐41 Trials,” JAMA Oncology 10, no. 5 (2024): 603–611. 38546612 10.1001/jamaoncol.2023.7304 PMC10979363

[31] S. Loibl , G. von Minckwitz , A. Schneeweiss , et al., “PIK3CA Mutations Are Associated With Lower Rates of Pathologic Complete Response to Anti‐Human Epidermal Growth Factor Receptor 2 (her2) Therapy in Primary HER2‐Overexpressing Breast Cancer,” Journal of Clinical Oncology 32, no. 29 (2014): 3212–3220. 25199759 10.1200/JCO.2014.55.7876

[32] B. Dave , I. Migliaccio , M. C. Gutierrez , et al., “Loss of Phosphatase and Tensin Homolog or Phosphoinositol‐3 Kinase Activation and Response to Trastuzumab or Lapatinib in Human Epidermal Growth Factor Receptor 2‐Overexpressing Locally Advanced Breast Cancers,” Journal of Clinical Oncology 29, no. 2 (2011): 166–173. 21135276 10.1200/JCO.2009.27.7814 PMC3058274

[33] G. Bianchini , A. Kiermaier , G. V. Bianchi , et al., “Biomarker Analysis of the NeoSphere Study: Pertuzumab, Trastuzumab, and Docetaxel Versus Trastuzumab Plus Docetaxel, Pertuzumab Plus Trastuzumab, or Pertuzumab Plus Docetaxel for the Neoadjuvant Treatment of HER2‐Positive Breast Cancer,” Breast Cancer Research 19, no. 1 (2017): 16. 28183321 10.1186/s13058-017-0806-9 PMC5299741

[34] M. F. Rimawi , C. De Angelis , A. Contreras , et al., “Low PTEN Levels and PIK3CA Mutations Predict Resistance to Neoadjuvant Lapatinib and Trastuzumab Without Chemotherapy in Patients With HER2 Over‐Expressing Breast Cancer,” Breast Cancer Research and Treatment 167, no. 3 (2018): 731–740. 29110152 10.1007/s10549-017-4533-9 PMC5821069

[35] F. Ma , Q. Li , S. Chen , et al., “Phase I Study and Biomarker Analysis of Pyrotinib, a Novel Irreversible Pan‐ErbB Receptor Tyrosine Kinase Inhibitor, in Patients With Human Epidermal Growth Factor Receptor 2‐Positive Metastatic Breast Cancer,” Journal of Clinical Oncology 35, no. 27 (2017): 3105–3112. 28498781 10.1200/JCO.2016.69.6179

[36] Q. Shi , J. Xuhong , T. Luo , et al., “PIK3CA Mutations Are Associated With Pathologic Complete Response Rate to Neoadjuvant Pyrotinib and Trastuzumab Plus Chemotherapy for HER2‐Positive Breast Cancer,” British Journal of Cancer 128, no. 1 (2023): 121–129. 36323880 10.1038/s41416-022-02021-z PMC9814131

[37] G. von Minckwitz , M. Procter , E. de Azambuja , et al., “APHINITY Steering Committee and Investigators. Adjuvant Pertuzumab and Trastuzumab in Early HER2‐Positive Breast Cancer,” New England Journal of Medicine 377, no. 2 (2017): 122–131. 28581356 10.1056/NEJMoa1703643 PMC5538020

[38] J. Bines , M. Procter , E. Restuccia , et al., “Incidence and Management of Diarrhea With Adjuvant Pertuzumab and Trastuzumab in Patients With Human Epidermal Growth Factor Receptor 2‐Positive Breast Cancer,” Clinical Breast Cancer 20, no. 2 (2020): 174–181. 31924513 10.1016/j.clbc.2019.06.016

[39] S. Hong , Y. Gu , Z. Gao , et al., “EGFR Inhibitor‐Driven Endoplasmic Reticulum Stress‐Mediated Injury on Intestinal Epithelial Cells,” Life Sciences 119, no. 1–2 (2014): 28–33. 25445223 10.1016/j.lfs.2014.10.008

[40] V. Hirsh , N. Blais , R. Burkes , S. Verma , and K. Croitoru , “Management of Diarrhea Induced by Epidermal Growth Factor Receptor Tyrosine Kinase Inhibitors,” Current Oncology 21, no. 6 (2014): 329–336. 25489260 10.3747/co.21.2241 PMC4257116

[41] A. Chan , M. Ruiz‐Borrego , G. Marx , et al., “Final Findings From the CONTROL Trial: Strategies to Reduce the Incidence and Severity of Neratinib‐Associated Diarrhea in Patients With HER2‐Positive Early‐Stage Breast Cancer,” Breast 67 (2023): 94–101. 36702070 10.1016/j.breast.2022.12.003 PMC9982309

[42] E. A. Eisenhauer , P. Therasse , J. Bogaerts , et al., “New Response Evaluation Criteria in Solid Tumours: Revised RECIST Guideline (Version 1.1),” European Journal of Cancer 45, no. 2 (2009): 228–247. 19097774 10.1016/j.ejca.2008.10.026

[43] J. W. Park , M. C. Liu , D. Yee , et al., “Adaptive Randomization of Neratinib in Early Breast Cancer,” New England Journal of Medicine 375, no. 1 (2016): 11–22. 27406346 10.1056/NEJMoa1513750 PMC5259558

