# Supplementary material for: Triple HER2 Blockade With Trastuzumab, Pertuzumab, and Pyrotinib Versus Dual HER2 Blockade in the Neoadjuvant Treatment of HER2‐Positive Breast Cancer: A Randomized, Phase II Study
Source: MedComm (2020). 2026 Jan 18;7(2):e70611. doi: 10.1002/mco2.70611 (PMC12812331; doi:10.1002/mco2.70611)

**Supplementary Information for: Triple HER2 blockade with trastuzumab, pertuzumab, and pyrotinib versus dual HER2 blockade in neoadjuvant treatment of HER2-positive breast cancer: a randomized, phase II study**

Jiahui Huang^#,1^, Haoyu Wang^#,1^, Yiwei Tong^#,1^, Jin Hong^#,1^, Yifei Zhu^1^, Weili Ren^2^, Jing Yu^1^, Haoting Shi^1^, Weiqi Gao^1^, Siji Zhu^1^, Jiayi Wu^1^, Ou Huang^1^, Jing Li^3^, Jianrong He^1^, Weiguo Chen^1^, Yafen Li^1^, Kunwei Shen^*,1^, Xiaosong Chen^*,1^

^#^These authors contributed equally to this work.
^1^ Department of General Surgery, Comprehensive Breast Health Center, Ruijin Hospital, Shanghai Jiao Tong University School of Medicine, Shanghai, China.

^2^ Department of Breast and Thyroid Surgery, Shangyu People’s Hospital, Shaoxing, Zhejiang, China

^3^ Department of Medical Affairs, Jiangsu Hengrui Pharmaceuticals Co., Ltd, Shanghai, China

* Corresponding to:

Xiaosong Chen, Email: chenxiaosong0156@hotmail.com; Address: Department of General Surgery, Comprehensive Breast Health Center, Ruijin Hospital, Shanghai Jiao Tong University School of Medicine, 197 Ruijin Er Road, Shanghai 200025, China

OR Kunwei Shen, Email: kwshen@medmail.com.cn; Address: Department of General Surgery, Comprehensive Breast Health Center, Ruijin Hospital, Shanghai Jiao Tong University School of Medicine, 197 Ruijin Er Road, Shanghai 200025, China

**Supplementary Tables**

**Table S1 Adjuvant treatment in study patients**

| Adjuvant Treatment  N (%) | tpCR | | non-tpCR | |
| --- | --- | --- | --- | --- |
|  | TPPy  *N* = 36 | TP  *N* = 32 | TPPy  *N* = 19 | TP  *N* = 21 |
| Chemotherapy |  |  |  |  |
| EC for 4cycles | 29 (80.6) | 26 (81.3) | 19 (100.0) | 20 (95.2) |
| nab-P for 2 cycles | 5 (9.4) | 4 (12.5) | 0 | 1 (4.8) |
| No further chemotherapy | 2 (3.8) | 2 (6.3) | 0 | 0 |
| Targeted Therapy |  |  |  |  |
| TP for 40 weeks | 36 (100.0) | 31 (96.9) | 13 (68.4) | 18 (85.7) |
| T-DM1 for 14 cycles | 0 | 0 | 3 (15.8) | 0 |
| T-DM1 for 14 cycles followed by neratinib for 1 year | 0 | 0 | 3 (15.8) | 1 (4.8) |
| TP with pyrotinib for 40 weeks | 0 | 0 | 0 | 1 (4.8) |
| No further targeted therapy | 0 | 1 (3.1) | 0 | 1 (4.8) |
| Radiation Therapy |  |  |  |  |
| YES | 34 (94.4) | 32 (100.0) | 18 (94.7) | 20 (95.2) |
| No | 2 (5.6) | 0 | 1 (5.3) | 1 (4.8) |
| Endocrine therapy |  |  |  |  |
| YES | 6 (16.7) | 7 (21.9) | 14 (73.7) | 15 (71.4) |
| Not Recommended | 30 (83.3) | 25 (78.1) | 5 (26.3) | 6 (28.6) |

TPPy, trastuzumab, pertuzumab and pyrotinib; TP, trastuzumab, pertuzumab; E, epirubicin, C, cyclophosphamide; Nab-P, nab-paclitaxel; T, trastuzumab; P, pertuzumab; Cb, carboplatin; Py, pyrotinib.

**Table S2 Clinical response in the TPPy and TP groups**

|  | TPPy group | TP group | P value |
| --- | --- | --- | --- |
|  | *N* = 55 | *N* = 53 |  |
| Clinical Response |  |  | 0.381 |
| CR | 31 (56.4) | 23 (43.4) |  |
| PR | 20 (36.4) | 26 (49.1) |  |
| SD | 4 (7.3) | 4 (7.5) |  |
| Overall Response Rate | 51 (92.7) | 49 (92.5) | 0.957 |

TPPy, trastuzumab, pertuzumab and pyrotinib; TP, trastuzumab, pertuzumab; CR, complete remission; PR, partial remission; SD, stable disease.

**Table S3 tpCR rate in patients in the TPPy group according to relative dose intensity quantile and median days on treatment**

|  | tpCR | non-tpCR | P value |
| --- | --- | --- | --- |
|  | *N* = 36 | *N* = 19 |  |
| Relative actual dose intensity (%)  Median 42.0 |  |  |  |
| < 21.1 (Q1) | 9 (64.3) | 5 (35.7) | 0.289 |
| 21.1 - 42.0 (Q2) | 12 (85.7) | 2 (14.3) |  |
| 42.0 - 75.0 (Q3) | 7 (53.8) | 6 (46.2) |  |
| ≥ 75.0 (Q4) | 8 (57.1) | 6 (42.9) |  |
| Days on treatment  Median 77 days |  |  |  |
| < 77 | 19 (65.5) | 10 (34.5) | 0.992 |
| ≥ 77 | 17 (65.4) | 9 (34.6) |  |

TPPy, trastuzumab, pertuzumab and pyrotinib; tpCR, total pathological complete response; Q, quartile.

**Table S4 Clinical studies on neoadjuvant pyrotinib.**

| Trial | Publication | Phase | Sample Size | Treatment | tpCR% |
| --- | --- | --- | --- | --- | --- |
| Xuhong et al | Oncologist, 2020 | II | 19 | ECPy×4-DTPy×4 | 73.7 |
| Shi et al | MedComm, 2023 | / | 175 | ECPy×4-DTPy×4 | 68.6 |
| NeoATP | Clin Cancer Res. 2022 | II | 53 | Paclitaxel-Cisplatin+TPy×4 | 69.8 |
| Panphila | Eur J Cancer, 2022 | II | 69 | DCbTPy×6 | 55.1 |
| Ding et al | Oncol Res Treat 2023 | II | 36  33 | DCbTPy×6  DCbT+placebo×6 | 65.5  33.3 |
| PHEDRA | BMC Med. 2022 | III | 178  177 | DTPy×4  DT+placebo×4 | 41.0  22.0 |

E, epirubicin, C, cyclophosphamide; D, docetaxel; T, trastuzumab; Py, pyrotinib; Cb, carboplatin; tpCR, total pathologic complete response rate.

**Table S5 Patient characteristics according to HER2 IHC scores.**

| Characteristics, N (%) | HER2 2+ | HER2 3+ | P value |
| --- | --- | --- | --- |
|  | *N* = 29 | *N* = 79 |  |
| Age |  |  | 0.686 |
| ≤50 years | 13 (44.8) | 32 (40.5) |  |
| >50 years | 16 (55.2) | 47 (59.5) |  |
| Menstruation |  |  | 0.868 |
| Postmenopause | 13 (44.8) | 34 (43.0) |  |
| Pre/Perimenopause | 16 (55.2) | 45 (57.0) |  |
| Clinical tumor stage |  |  | 0.064 |
| 0-2 | 13 (44.8) | 51 (64.6) |  |
| 3-4 | 16 (55.2) | 28 (35.4) |  |
| Clinical node stage |  |  | 0.253 |
| 0 | 0 (0.0) | 9 (11.4) |  |
| 1 | 7 (24.1) | 14 (17.7) |  |
| 2 | 12 (41.4) | 34 (43.0) |  |
| 3 | 10 (34.5) | 22 (27.8) |  |
| Clinical AJCC stage |  |  | 0.161 |
| II | 2 (6.9) | 18 (22.8) |  |
| IIIA | 15 (51.7) | 36 (45.6) |  |
| IIIB-C | 12 (41.4) | 25 (31.6) |  |
| Estrogen receptor status |  |  | <0.001 |
| Positive | 19 (65.5) | 21 (26.6) |  |
| Negative | 10(34.5) | 58 (73.4) |  |
| Progesterone receptor status |  |  | <0.001 |
| Positive | 14 (48.3) | 12 (15.2) |  |
| Negative | 15 (51.7) | 67 (84.8) |  |
| Hormone receptor status |  |  | 0.001 |
| Positive | 19 (65.5) | 24 (30.4) |  |
| Negative | 10 (34.5) | 55 (69.6) |  |
| Ki67, % |  |  | 0.207 |
| ≤30 | 10 (34.5) | 38 (48.1) |  |
| >30 | 19 (65.5) | 41 (51.9) |  |

HER2, human epidermal growth factor receptor 2; IHC, immunohistochemistry; AJCC, American Joint Committee on Cancer

**Table S6 PI3K/AKT/mTOR pathway analyses related with pCR in TPPy and TP group**

| Pathways | TPPy group | | TP group | |
| --- | --- | --- | --- | --- |
|  | NES | Adjusted p | NES | Adjusted p |
| Phosphatidylinositol 3 kinase signaling | 0.91 | 0.98 | 1.63 | 0.07 |
| Regulation of phosphatidylinositol 3 kinase signaling | 1.27 | 0.98 | 1.23 | 0.20 |
| Positive regulation of phosphatidylinositol 3 kinase signaling | 0.79 | 0.99 | 1.23 | 0.20 |
| mTOR signaling pathway | 4.14 | 0.42 | 7.14 | 0.22 |
| Phosphatidylinositol signaling system | 1.20 | 0.98 | 12.28 | 0.09 |

TPPy, trastuzumab, pertuzumab and pyrotinib; TP, trastuzumab, pertuzumab; NES, normalized enrichment scores.

**Table S7 GSEA pathway analyses related with pCR in TPPy and TP group**

| **Pathways** | **TPPy group** | | | **TP group** | | |
| --- | --- | --- | --- | --- | --- | --- |
|  | NES | Adjusted p | FDR q | NES | Adjusted p | FDR q |
| Adaptive immune response | 3.79 | 8.95E-07 | 8.95E-07 | 5.74 | 1.17E-24 | 9.64E-25 |
| Immune response | 3.03 | 0.001 | 0.001 | 5.81 | 5.86E-24 | 4.82E-24 |
| B cell mediated immunity | 2.68 | 0.001 | 0.001 | 3.50 | 1.39E-08 | 1.14E-08 |
| T cell activation | 2.43 | 0.023 | 0.023 | 2.14 | 0.029 | 0.024 |
| Carbohydrate metabolism | 1.7 | 0.023 | 0.023 | 1.27 | 0.201 | 0.165 |
| Regulate of GTPase activity | 1.65 | 0.023 | 0.023 | 1.28 | 0.201 | 0.165 |
| Oxidative Phosphorylation | 1.58 | 0.033 | 0.033 | 1.21 | 0.323 | 0.265 |
| Glycoprotein Biosynthetic Process | 1.61 | 0.042 | 0.042 | 1.07 | 0.386 | 0.317 |
| Phagocytosis | 1.48 | 0.976 | 0.976 | 2.58 | 7.52E-05 | 6.18E-05 |
| Endocytosis | 1.43 | 0.976 | 0.976 | 3.07 | 2.11E-06 | 1.73E-06 |
| Phagocytosis Recognition | 1.29 | 0.976 | 0.976 | 2.58 | 7.52E-05 | 6.18E-05 |

GSEA, gene set enrichment analysis; TPPy, trastuzumab, pertuzumab and pyrotinib; TP, trastuzumab, pertuzumab; NES, normalized enrichment scores; FDR, false discovery rate.

**Supplementary Figure Legends**

**Figure S1. Bayesian estimation at interim analysis.** The tpCR rate was estimated to be 65.8% (95% PI, 50.7 to 68.9%) in TPPy group, and 60.2% (95% PI, 56.4 to 74.0%) in TP group. The predictive probability of superior was 7% and the probability of equivalence was 92.9% in full planned enrollment.

TPPy, trastuzumab, pertuzumab and pyrotinib; TP, trastuzumab, pertuzumab; tPCR, total pathological complete remission; PI, predictive interval.

**Figure S2. Recurrence-free survival comparison between treatment arms.**

TPPy, trastuzumab, pertuzumab and pyrotinib; TP, trastuzumab, pertuzumab.

**Figure S3. KEGG pathways and immune cell infiltration correlated with pCR.** (A) Heatmap demonstrating KEGG pathways exclusively related with pCR in TPPy group and TP group. Upregulated alpha linoleic acid metabolism (unadjusted P = 0.027), linoleic acid metabolism (unadjusted P = 0.021) and ether lipid metabolism (unadjusted P = 0.043) was correlated with higher likelihood of pCR in TPPy group; On the other hand, enriched pathways of toll-like receptor signaling (unadjusted P = 0.032), Fcε RI signaling (unadjusted P = 0.046), Regulation of autophagy (unadjusted P = 0.016) and Histidine metabolism (unadjusted P = 0.022) were significantly related with higher pCR rate in TP group. (B) Violin plot showing immune cells predictive for pathological response in total population.

TPPy, trastuzumab, pertuzumab and pyrotinib; TP, trastuzumab, pertuzumab; KEGG, Kyoto Encyclopedia of Genes and Genomes.

**Figure S4 PAM50 intrinsic subtypes according to HER2 IHC scores.**

HER2, human epidermal growth factor receptor-2; IHC, immunohistochemistry.

**Supplementary Figures**

**Figure S1**


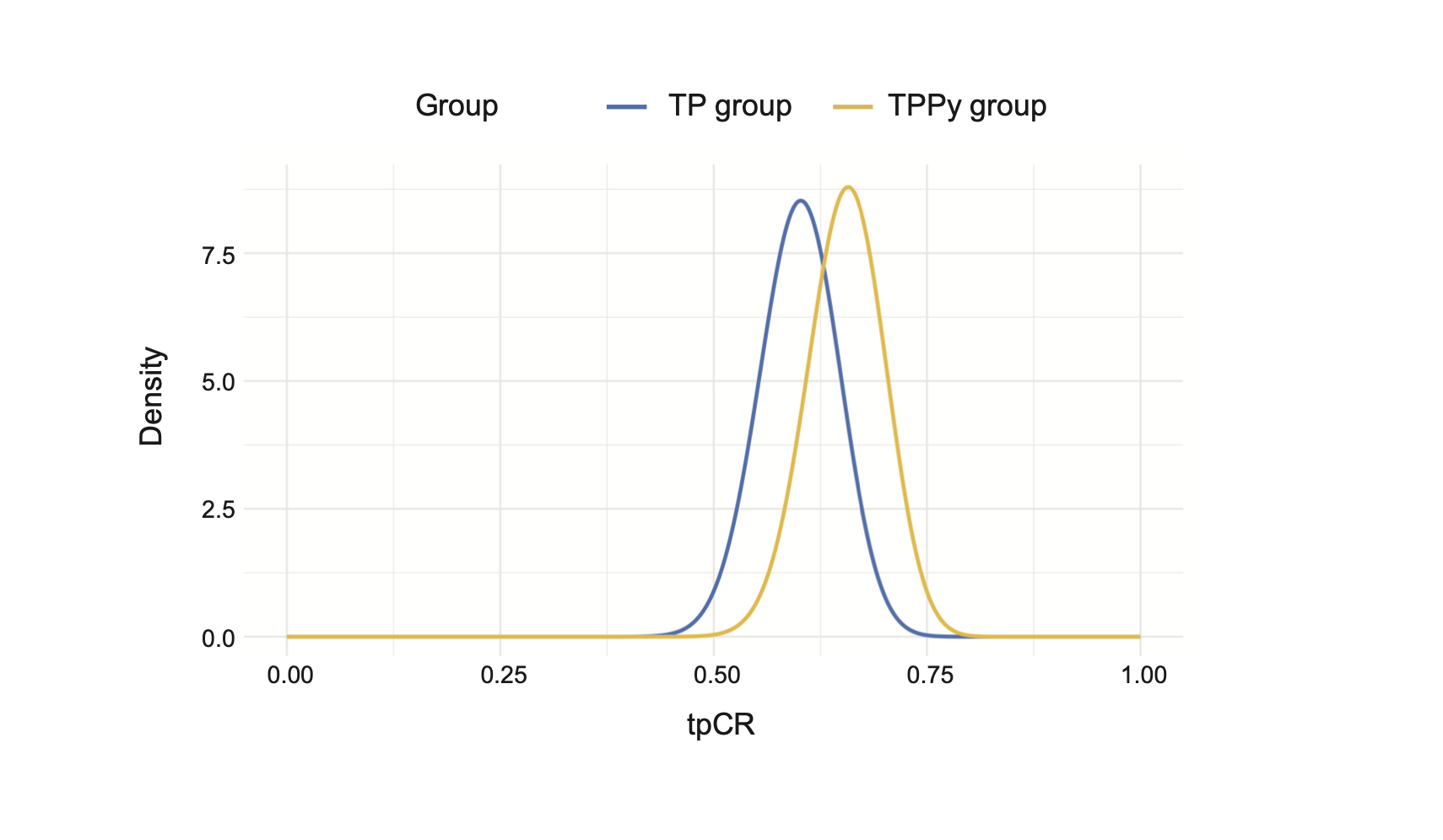


**Figure S2**


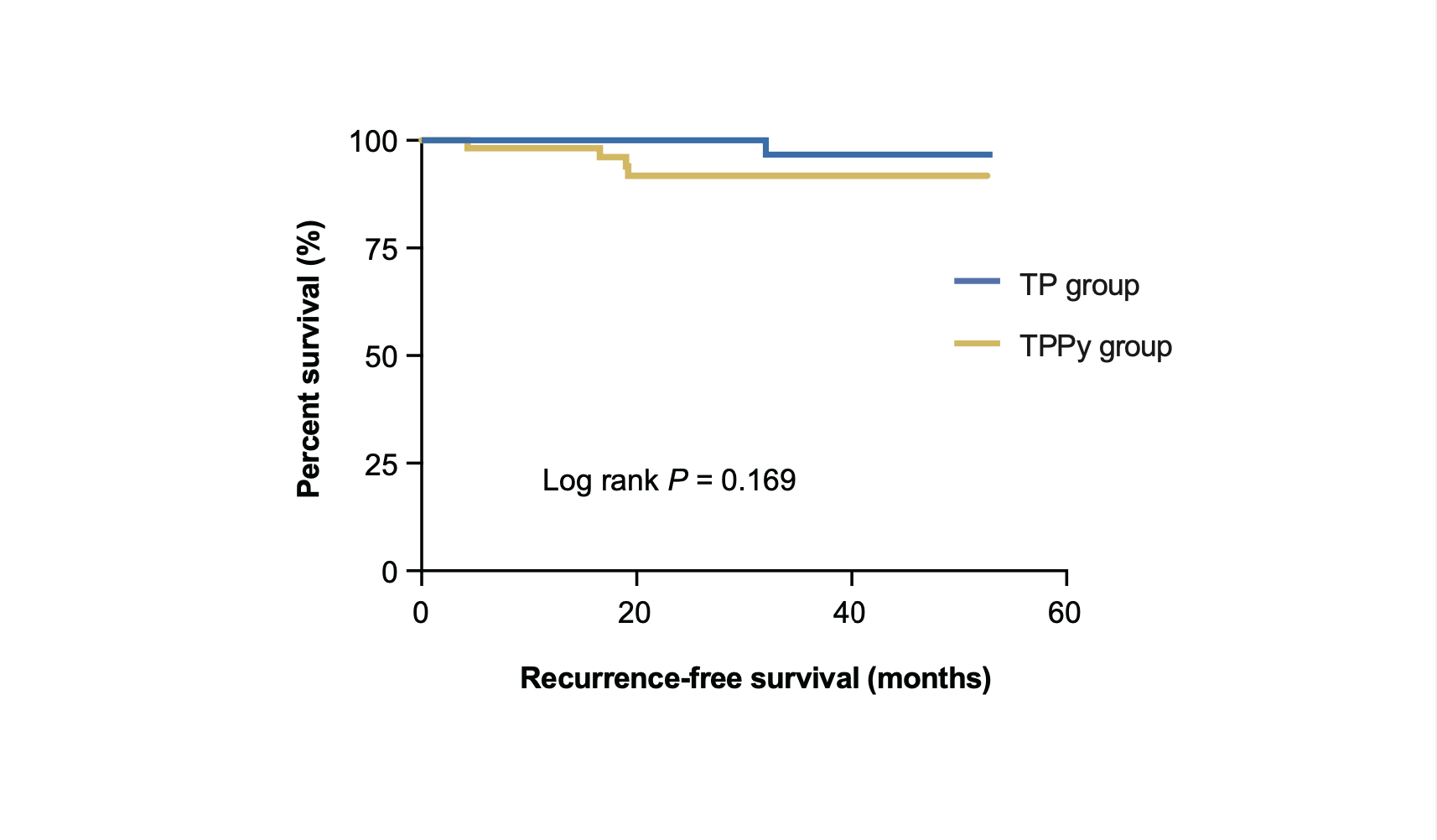


**Figure S3**


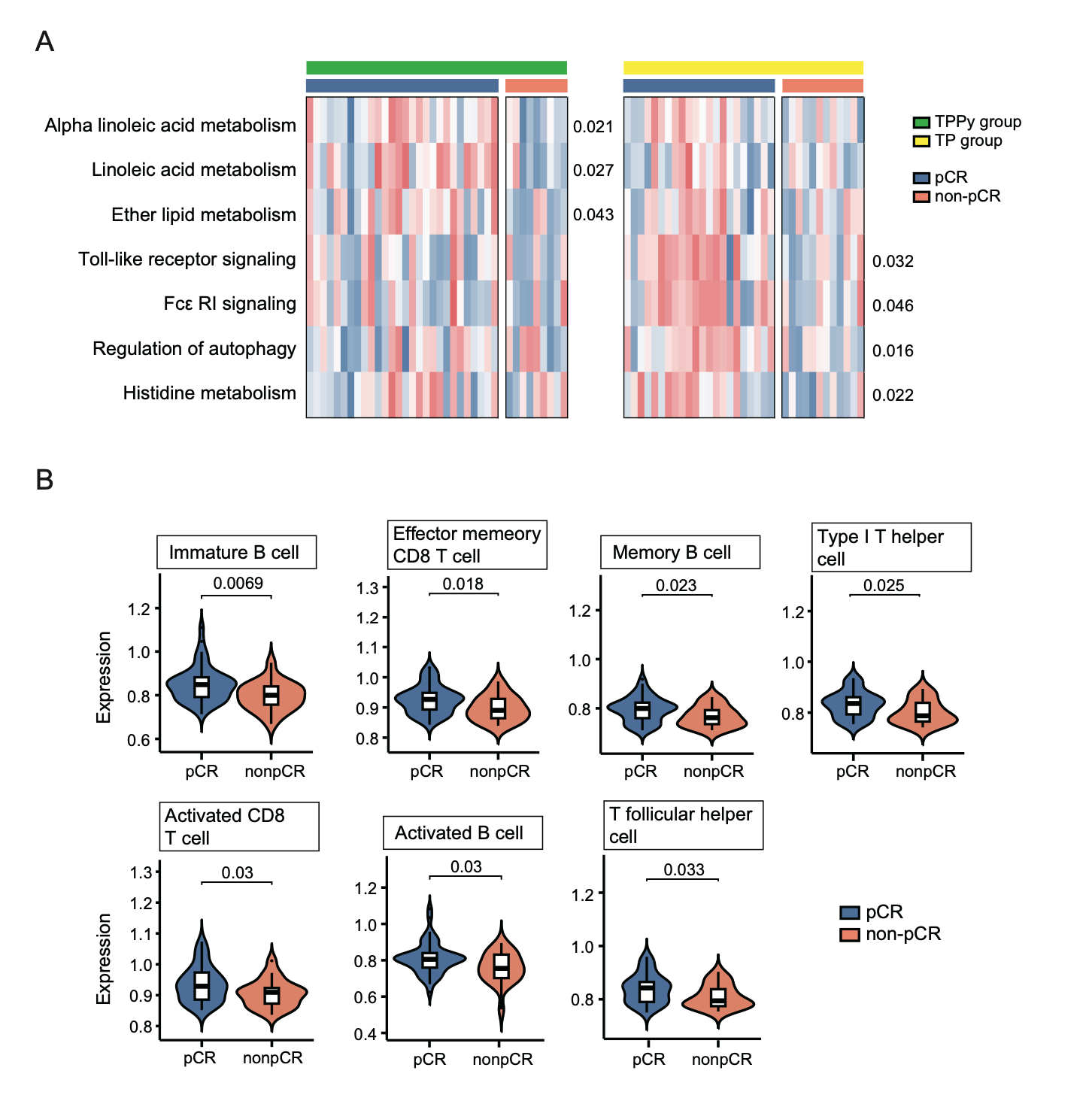


**Figure S4**


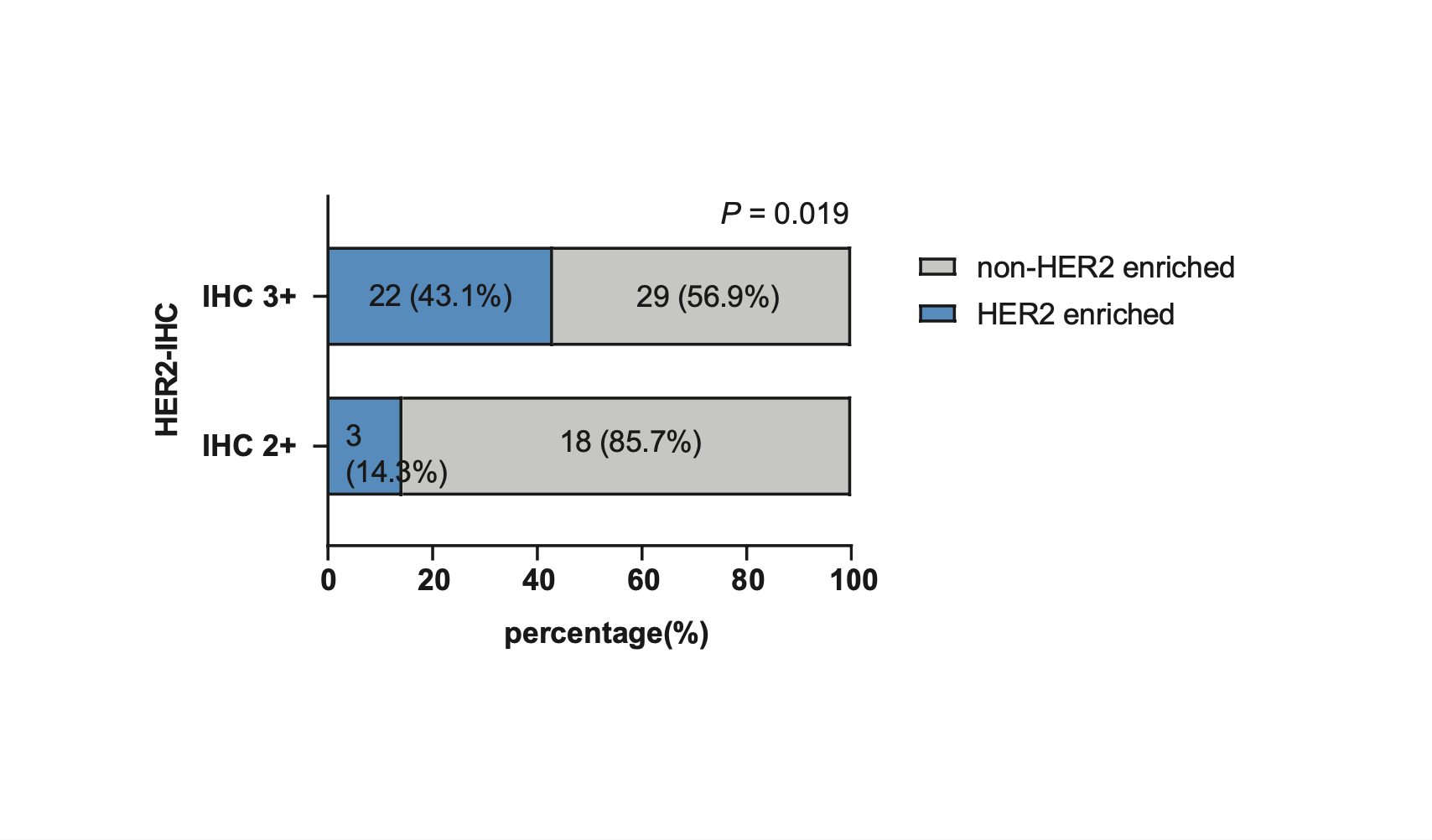

Supplement: Supplementary file 1 — Table S1 Adjuvant treatment in study patients. S2 Clinical response in the TPPy and TP groups. Table S3 tpCR rate in patients in the TPPy group according to relative dose intensity quantile and median days on treatment. Table S4 Clinical studies on neoadjuvant pyrotinib. Table S5 Patient characteristics according to HER2 IHC scores. Table S6 PI3K/AKT/mTOR pathway analyses related with pCR in TPPy and TP group. Table S7 GSEA pathway analyses related with pCR in TPPy and TP group. Figure S1. Bayesian estimation at interim analysis. The tpCR rate was estimated to be 65.8% (95% PI, 50.7 to 68.9%) in TPPy group, and 60.2% (95% PI, 56.4 to 74.0%) in TP group. The predictive probability of superior was 7% and the probability of equivalence was 92.9% in full planned enrollment. TPPy, trastuzumab, pertuzumab and pyrotinib; TP, trastuzumab, pertuzumab; tPCR, total pathological complete remission; PI, predictive interval. Figure S2. Recurrence‐free survival comparison between treatment arms. TPPy, trastuzumab, pertuzumab and pyrotinib; TP, trastuzumab, pertuzumab. Figure S3. KEGG pathways and immune cell infiltration correlated with pCR. (A) Heatmap demonstrating KEGG pathways exclusively related with pCR in TPPy group and TP group. Upregulated alpha linoleic acid metabolism (unadjusted P = 0.027), linoleic acid metabolism (unadjusted P = 0.021) and ether lipid metabolism (unadjusted P = 0.043) was correlated with higher likelihood of pCR in TPPy group; On the other hand, enriched pathways of toll‐like receptor signaling (unadjusted P = 0.032), Fcε RI signaling (unadjusted P = 0.046), Regulation of autophagy (unadjusted P = 0.016) and Histidine metabolism (unadjusted P = 0.022) were significantly related with higher pCR rate in TP group. (B) Violin plot showing immune cells predictive for pathological response in total population. TPPy, trastuzumab, pertuzumab and pyrotinib; TP, trastuzumab, pertuzumab; KEGG, Kyoto Encyclopedia of Genes and Genomes. Figure S4 PAM50 intrinsic s [file MCO2-7-e70611-s001.docx]
